# Long term outcome after respiratory ecmo and length of ecmo treatment

**DOI:** 10.1186/2197-425X-3-S1-A153

**Published:** 2015-10-01

**Authors:** H Kalzén, V von Bahr, K Palmer, B Frenckner

**Affiliations:** Karolinska University Hospital, Solna, ECMO ICU, Stockholm, Sweden

## Introduction

Respiratory ECMO is nowadays a common tool in the ICU to help the patient survive despite severe respiratory distress and gas exchange problems. The pathology can be diverse but the most common diagnoses are pneumonia/ARDS. The treatment time depends on several factors, including the underlying pathology, patient characteristics and complications.

## Objectives

To investigate how long term survival for adults after respiratory ECMO correlates to time on ECMO.

## Methods

A consecutive cohort of all adults, n = 273, treated in the ECMO ICU of Karolinska University Hospital Stockholm Sweden between 1995-12-15 to 2013-12-30 and followed up to 2014-01-15 was used(1). Data on length of stay on ECMO, and long-term outcome up to five years post ECMO treatment, was extracted. Six different groups was formed depending on length of ECMO treatment. Outcome after ICU and Hospital discharge, 90 days and 5 years was analysed. Alive after 90 days and still alive five years later was also analysed.

## Results

The highest survival, 74%, was seen in the group with the shortest stay,1-7 days on ECMO. Outcome in this group dropped to 56% survival after 5 years of follow up. With longer ECMO stay, outcome generally, but not linearly, declined and was still 47% ICU survival in the group with up to 56 days on ECMO. The group >56 days of ECMO treatment, containing only six patients, had a 33% ICU outcome. Interestingly in four out of six groups the patients being alive after 90 days had a 75-100% 5 year survival post ECMO treatment.

## Conclusions

ICU outcome after respiratory ECMO in this cohort study was between 33-74% depending on length of stay on ECMO. Best outcome was seen in the groups with the shortest length of stay. With longer ECMO stay, outcome generally, but not linearly, declined. Interestingly the group being alive 90 days post ECMO had a 5 years survival between 75-100% The findings though need to be carefully interpereted since some of the groups are very small.

**Table 1:** Time on ECMO and long term outcome.

Time on ECMO days	n= (total n = 273)	Alive from ECMO ICU	Alive at Hospital discharge	Alive after 90 days	Alive after 5 years	Alive after 5 years if alive after 90 days
1-7 d	127	94 (74%)	91 (72%)	73 (59%)	36/64 (56%)	28/35 (80%)
8-14 d	72	53 (74%)	51 (72%)	44/68 (65%)	15/31 (48%)	15/17 (88%)
15-21 d	22	12 (55%)	12 (55%)	8 (36%)	2/8 (25%)	2/2 (100%)
22-28 d	17	12 (71%)	12 (71%)	9 (53%)	6/12 (50%)	6/8 (75%)
29-42 d	12	5 (42%)	5 (42%)	5 (42%)	0/1 (0%)	0/0 (0%)
43-56 d	17	8 (47%)	8 (47%)	8 (47%)	3/9 (33%)	4/4 (100%)
> 56 d	6	2 (33%)	2 (33%)	2 (33%)	0/1 (0%)	0/0 (0%)
